# Spectrum of Viral Pathogens in Blood of Malaria-Free Ill Travelers Returning to Canada

**DOI:** 10.3201/eid2205.151875

**Published:** 2016-05

**Authors:** Ruwandi Kariyawasam, Rachel Lau, Alireza Eshaghi, Samir N. Patel, Doug Sider, Jonathan B. Gubbay, Andrea K. Boggild

**Affiliations:** Public Health Ontario Laboratories, Toronto, Ontario, Canada (R. Kariyawasam, R. Lau, A. Eshaghi, S.N. Patel, J.B. Gubbay, A.K. Boggild);; University of Toronto, Toronto (S.N. Patel, J.B. Gubbay, A.K. Boggild);; Public Health Ontario, Toronto (D. Sider);; McMaster University, Hamilton, Ontario, Canada (D. Sider);; Toronto General Hospital, Toronto (A.K. Boggild)

**Keywords:** fever in returned travelers, Flaviviridae, flaviviruses, Togaviridae, travel medicine, tropical medicine, viruses, Canada, pathogens, travel-acquired viral pathogens, viruses, dengue virus, chikungunya virus, vectorborne infections, imported viral infections

## Abstract

Malaria is the most common specific cause of fever in returning travelers, but many other vectorborne infections and viral infections are emerging and increasingly encountered by travelers. We documented common and emerging viral pathogens in malaria-negative specimens from ill travelers returning to Canada. Anonymized, malaria-negative specimens were examined for various viral pathogens by real-time PCR. Samples were positive for herpes simplex viruses 1 or 2 (n = 21, 1.6%), cytomegalovirus (n = 4, 0.3%), Epstein-Barr virus (n = 194, 14.9%), dengue virus types 1–4 (n = 27, 2.1%), chikungunya virus (n = 5, 0.4%), and hepatitis A virus (n = 12, 0.9%). Travel-acquired viral pathogens were documented in >20% of malaria-negative specimens, of which 2.5% were infected with dengue and chikungunya viruses. Our findings support the anecdotal impression that these vectorborne pathogens are emerging among persons who travel from Canada to other countries.

## Abstract 2

Over a 2-year period, common and emerging viruses were documented in >20% of these travelers.

Malaria is the most common specific cause of fever in travelers returning from tropical or subtropical travel; however, many vectorborne and viral infections are emerging in new geographic areas and are increasingly encountered by persons who travel. In multi- and single-center analyses, the top specific causes of fever in returning travelers include malaria (20%–30%), acute travelers’ diarrhea or gastroenteritis (10%–20%), respiratory tract infections (10%–15%), dengue fever (5%–10%), enteric fever due to *Salmonella enterica* serotypes Typhi and Paratyphi (2%–7%), skin and soft tissue infections (2%–11%), rickettsioses (3%), acute urinary tract and sexually transmitted infections (2%–3%), viral hepatitis (3%), and mononucleosis-like syndrome (3%–25%) (*1*–*6*).

In North America, the burden of imported notifiable diseases, such as malaria (*7*) enteric fever (*8*), influenza (*9*), measles (*10*), and viral hepatitis (*9*), is reasonably well documented, but emerging imported viral infections, such as dengue virus (DENV) and chikungunya virus (CHIKV) infections, are not necessarily reportable to public health agencies (*11*). Thus, the anecdotal impression among clinicians that these illnesses are increasingly being imported is difficult to substantiate because national level statistics are lacking. Annual surveillance reports by CanTravNet indicate that before the establishment of CHIKV in the Americas in late 2013, cases of CHIKV infection were infrequently reported among travelers, but infections that did occur were mostly imported from the Indian Ocean islands and Southeast Asia (*12*,*13*). Mathematical modeling of infectious diseases predicts that importations of CHIKV infections to areas where the disease is not endemic are probably vastly underestimated and on the rise (*14*).

Moreover, as noted above, a high percentage of febrile returned travelers are ultimately diagnosed with a nonspecific viral syndrome, which tends to resolve uneventfully without therapy and remains unconfirmed by specific diagnostic tests. In our recent GeoSentinel network–wide analysis of illness in travelers returning from Sierra Leone, Liberia, or Guinea (*15*), we noted that fever was the most common reason for seeking healthcare, and the fever was most commonly due to malaria. However, “nonspecific viral syndrome” and “febrile illness not otherwise specified lasting <3 weeks” were the fourth and fifth most common diagnoses made for ill returned nonimmigrant travelers (*15*). Similarly, other studies have noted that up to 40% of febrile returned travelers receive no specific etiologic diagnosis (*16*–*18*). Thus, a substantial diagnostic gap exists, considering that fever in returning travelers may herald life-threatening, emerging infections of critical public health importance. To better understand the epidemiology of imported viral infections, we documented the spectrum of common and emerging viral pathogens in ill travelers returning to Canada who did not have malaria.

## Methods

### Specimens

The malaria biobank at the Public Health Ontario Laboratory (Toronto, ON, Canada) houses aliquots of denominalized, surplus whole blood (EDTA) specimens submitted for malaria diagnostic testing; specimens are maintained at −80°C. The biobank database contains denominalized demographic and clinical data (e.g., age, sex, and travel history) provided on test requisition forms as well as parasitologic data (e.g., parasitemia and malaria species) for positive specimens.

For this study, we used anonymized, surplus EDTA-containing specimens stored in the malaria biobank during May 2006–April 2007 and anonymized malaria-negative whole blood specimens that were accrued prospectively during February 2013–March 2014. Ethics approval was obtained from the Public Health Ontario Ethics Review Board. Extracted data were limited to age, sex, and travel history when provided on the standard test requisition form and included in the malaria biobank.

Before sending negative specimens to the malaria biobank, we performed routine malaria diagnostics in the clinical laboratory; these diagnostics were performed by microscopy examination of Giemsa-stained thick and thin blood films and by using the rapid diagnostic test with the BinaxNOW Malaria test kit (Alere, Scarborough, ME, USA). We confirmed all malaria results at study initiation using a multiplex real-time PCR assay (*19*). A malaria-negative specimen was defined as one for which Giemsa-stained expert microscopy and rapid diagnostic test results were negative and the patient did not have a documented prior episode of malaria in the past year. All malaria-negative specimens accrued during the enrollment period were subjected to a series of real-time PCR assays, as described in the following sections. We considered these specimens to have come from febrile or ill returned travelers because malaria is not endemic in Canada and, thus, testing for the disease necessarily implies illness after international tropical or subtropical travel. Fever is the most common complaint at the initial healthcare visit for persons diagnosed with malaria (*18*).

### DNA and RNA Extraction from Whole Blood

We used the KingFisher Flex 96 Instrument (Thermo Fisher Scientific, Waltham, MA, USA) according to the manufacturer’s instructions to separately perform DNA and RNA extractions from anonymized malaria-negative specimens. To extract DNA, we used the KingFisher Pure Blood Kit (Thermo Fisher Scientific), with the following modifications to volume requirements: 150 μL of EDTA blood, 75 µL of lysis buffer, and 15 µL of proteinase K. To extract RNA, we used the KingFisher Pure Viral NA Kit (Thermo Fisher Scientific) with the following modifications to volume requirements: 150 µL of EDTA blood, 161 µL of lysis buffer, 4 µL of carrier RNA, 40 µL of proteinase K, and 15 µL of exogenous internal control MS2 (final concentration 1 × 10^4^ copies/µL). Purified DNA and RNA elutes from each specimen were collected from the elution plate and made into two 50-μL aliquots for working (−20°C) and storage (−80°C) stocks.

### Control Specimens

As positive controls, we used human DNA; MS2 (Roche Applied Sciences, Laval, QC, Canada); *Plasmodium falciparum* and *P. vivax* small subunit rRNA DNA clones (MRA-177 and MRA-178, MR4; ATCC, Manassas VA, USA); CHIKV MBC099 AmpliRun PCR Controls (Phoenix Airmid Biomedical, Oakville, ON, Canada). We also used previously characterized in-house, positive controls (i.e., herpes simplex virus [HSV]-1 and HSV-2, cytomegalovirus [CMV], Epstein-Barr virus [EBV], DENV serotypes 1–4 [DENV-1–4], and hepatitis A virus [HAV]).

### Detection of Viral DNA by Real-Time PCR

We used real-time PCR with previously published primers and probes (Table 1) to test all specimens for viral DNA. A total volume of 25 µL was used; the volume included 12.5 µL of TaqMan Universal MasterMix (Life Technologies, Carlsbad, CA, USA) and 5 µL of template DNA for the *Plasmodium* genus and B2MG assay as well as 3 µL of template DNA for HSV-1, HSV-2, EBV, and CMV assays. All reactions were performed on the Applied Biosystems 7500 Fast Real-Time PCR System (Thermo Fisher Scientific), using the following conditions: 2 min at 50°C, 10 min at 95°C, 45 cycles of 15 sec at 95°C, and 1 min at 60°C, according to the manufacturer’s protocol. A specimen with a cycle threshold (C_t_) of <40 in the presence of a logarithmic amplification curve was considered positive for a viral pathogen.

**Table 1:** Primers and probes used to test specimens from ill travelers returning to Canada for viral DNA*

Primer and probe	Sequence, 5′ → 3′	Concentration, nmol/L	Reference
Human B2MG			(*20*)
B2MG fwd primer	TGAGTATGCCTGCCGTGTGA	900	
B2MG rev primer	ACTCATACACAACTTTCAGCAGCTTAC	900	
B2MG probe	HEX-CCATGTGACTTTGTCACAGCCCAAGATAGTT-BHQ1	200	
*Plasmodium*			(*21*)
Plasmo1 primer	GTTAAGGGAGTGAAGACGATCAGA	200	
Plasmo2 primer	AACCCAAAGACTTTGATTTCTCATAA	200	
Plasmo probe	FAM-ACCGTCGTAATCTTAACCATAAACTATGCCGACTAG-BHQ1	50	
HSV-1 and -2			(*22*)
GbTypF primer	CGCATCAAGACCACCTCCTC	600	
GbTypR primer	GCTCGCACCACGCGA	600	
GbTyp1 probe	VIC-TGGCAACGCGGCCCAAC-TAMRA	200	
GbTyp2probe	FAM-CGGCGATGCGCCCCAG-TAMRA	200	
CMV			(*23*)
CMV IE fwd primer	GACTAGTGTGATGCTGGCCAAG	500	
CMV IE rev primer	GCTACAATAGCCTCTTCCTCATCTG	500	
CMV IE probe	HEX-AGCCTGAGGTTATCAGTGTAATGAAGCGCC-BHQ1	125	
EBV			(*23*)
EBV-BALF fwd primer	CGGAAGCCCTCTGGACTTC	500	
EBV-BALF5 rev primer	CCCTGTTTATCCGATGGAATG	500	
EBV BALF5 probe	FAM-TGTACACGCACGAGAAATGCGCC-BHQ1	125	

*B2MG, β2 microglobulin; CMV, cytomegalovirus; EBV, Epstein-Barr virus; fwd, forward; HSV, herpes simplex virus; rev, reverse.

### Detection of Viral RNA by Real-Time Reverse Transcription PCR (RT-PCR)

To test specimens for the presence of viral RNA, we used real-time RT-PCR with previously published primers and probes for dengue-MS2, panflavivirus, HAV, and CHIKV (Table 2). We used the reverse complement of ChikR10487-10508 (5′-GGTGTCCAGGCTGAAGACATTG-3′) because the sense sequence was published for the reverse primer in the CHIKV assay (*28*). A total volume of 20 µL was used, including 5 µL of TaqMan Fast Virus 1-Step MasterMix (Life Technologies, Burlington, ON, Canada) and 3 µL of template RNA. We performed all reactions on the Applied Biosystems 7500 Fast Real-Time PCR System according to the manufacturer’s protocol, using the following conditions: 5 min at 50°C, 20 sec at 95°C, 45 cycles of 3 sec at 95°C, and 30 sec at 60°C. A specimen with a C_t_ <40 in the presence of a logarithmic amplification curve was considered positive for a viral pathogen.

**Table 2:** Primers and probes used to test specimens from ill travelers returning to Canada for viral RNA*

Primer and probe	Sequence, 5′→ 3′	Concentration, nmol/L	Reference
Pandengue-MS2			(*23*,*24*)
Den Eili fwd primer	GGACTAGAGGTTAGAGGAGACCCC	500	
Den Eili rev primer	GAGACAGCAGGATCTCTGGTC	500	
Den Eili probe	FAM-AGCATATTGACGCTGGGA-MGB-BHQ1	125	
MS2-TM2 fwd primer	TGCTCGCGGATACCCG	500	
MS2-TM2 rev primer	AACTTGCGTTCTCGAGCGAT	500	
MS2-TM2 probe	Quasar670-ACCTCGGGTTTCCGTCTTGCTCGT-BHQ1	125	
Dengue virus serotyping			(*25*)
DENV-1 fwd primer	CAAAAGGAAGTCGTGCAATA	500	
DENV-1 rev primer	CTGAGTGAATTCTCTCTACTGAACC	500	
DENV-1 probe	FAM-CATGTGGTTGGGAGCACGC-BHQ1	125	
DENV-2 fwd primer	CAGGTTATGGCACTGTCACGAT	500	
DENV-2 rev primre	CCATCTGCAGCAACACCATCTC	500	
DENV-2 probe	HEX-CTCTCCGAGAACAGGCCTCGACTTCAA-BHQ-1	125	
DENV-3 fwd primer	GGACTGGACACACGCACTCA	500	
DENV-3 rev primer	CATGTCTCTACCTTCTCGACTTGTCT	500	
DENV-3 probe	FAM-ACCTGGATGTCGGCTGAAGGAGCTTG-BHQ1	125	
DENV-4 fwd primer	TTGTCCTAATGATGCTGGTCG	500	
DENV-4 rev primer	TCCACCTGAGACTCCTTCCA	500	
DENV-4 probe	HEX-TTCCTACTCCTACGCATCGCATTCCG-BHQ1	125	
Panflavivirus			(*26*)
Flavi all S (sense)	TACAACATgATggggAARAgAgARAA	500	
Flavi all AS2 (antisense)	gTgTCCCAgCCNgCKgTgTCATCWgC	500	
DENV-4F	TACAACATgATgggRAAACgTgAGAA	500	
Flavi all probe 1	FAM-AARggHAgYMgNgCCA+TH+T+g+g+T-BBQ†	100	
Flavi probe 3a	FAM-CCgTgCCATATggTATATgTggCTgggAgC-BBQ†	100	
Flavi probe 3b	FAM-TTTCTggAATTTgAAgCCCTgggTTT-BBQc	100	
Hepatitis A virus			(*27*); vital SOP‡
HAV 68	TCACCGCCGTTTGCC	500	
HAV240	GGAGAGCCCTGGAAGAAAG	500	
HAV150 probe	FAM-CCTGAACCTGCAGGAATTAA-MGB-NFQ	125	
Chikungunya virus			(*28*)
ChikF10378–10398	GCATCAGCTAAGCTCCGGGTC	500	
ChikR 10487–10508¶	GGTGTCCAGGCTGAAGACATTG	500	
Chik Pongsiri	HEX-ATGCAAACGGCGACCATGCCGTCA-VIC	125	

*The primers and probes were used with the Applied Biosystems 7500 Fast Real-Time PCR System (Thermo Fisher Scientific, Waltham, MA, USA). fwd, forward; rev, reverse; SOP, standard operating procedure. †Locked nucleic acid bases. ‡Vital Standard Operating Procedure modified from Costafreda et al. (*27*). ¶ChikR10487-10508 reverse complement used from published sequence.

#### Dengue and Panflavivirus Real-Time RT-PCR Assays

A total of 5 µL of real-time RT-PCR product with positive signal (i.e., logarithmic amplification curve) from the DENV or panflavivirus assay were loaded onto a 1% agarose gel and visualized under UV light for any bands. These samples were then used for subsequent sequencing. Using sequence alignment and BLAST searching, we verified that the panflavivirus primers and probes should detect the following viruses, if present in sufficient quantities in a specimen: DENV-1–4; yellow fever (strains 17D, Asibi, Brazil, and Ivory Coast); West Nile virus (strains from Uganda and Israel); tickborne encephalitis virus (strains k23 and Louping ill); Japanese, St. Louis, and Murray Valley encephalitis viruses; and Wesselsbron, Zika, Yaounde, and Nounane viruses.

#### Dengue Serotype Determination by Duplex Real-Time RT-PCR Assays

Before sequencing pan-DENV– and panflavivirus-positive samples, we performed second confirmatory assays on all positive specimens to detect all 4 DENV serotypes; the assays consisted of 2 duplex real-time RT-PCRs targeting DENV-1/2 and DENV-3/4, respectively (Table 2). Two separate duplex reactions were performed with a volume of 25 µL. The first reaction used 12.5 µL of QuantiTect Probe RT-PCR MasterMix (QIAGEN, Germantown, MD, USA); 500 nM primers DENV-1JF, DENV-1JR, DENV-2JF, and DENV-2JR; 125 nM probes 1Jpr, DENV-2Jpr, DENV-3Jpr, and DENV-4Jpr; and 5 µL of template RNA. The second reaction used 12.5 µL of QuantiTect Probe RT-PCR MasterMix; 500 nM primers DENV-3JF, DENV-3JR, DENV-4JF, and DENV-4JR; 125 nM probes DENV-3Jpr and DENV-4Jpr; and 5µL of template RNA. We used the Applied Biosystems 7500 Fast Real-Time PCR System with the following cycling conditions: 30 min at 50°C, 15 min at 95°C, 45 cycles of 15 sec at 94°C, and 1 min at 60°C.

### Sequencing and Analysis

We performed Sanger sequencing reactions using the Big Dye v3.1 Cycle Sequencing Kit (Life Technologies, Carlsbad, CA, USA). For each cycle sequencing reaction, we used a 20-µL volume, including 1 µL of PCR product, 2 µL of Big Dye, 3 µL of buffer, and 2 µL of 10 µM primer. We used a C1000 Touch Thermal Cycler (Bio-Rad, Hercules, CA, USA) according to the manufacturer’s instructions and under the following cycling conditions: 1 min at 96°C, 25 cycles of 10 sec at 96°C, 5 sec at 50°C, and 4 min at 60°C. We then cleaned the product, using the DyeEx 2.0 Spin Kit (QIAGEN), and placed it into a speed vacuum for 35 min, including 10 min of heat. Next, we added 20 µL of Hi-Di Formamide (Thermo Fisher Scientific) to each sample and performed sequencing using the ABI 3730xl DNA Analyzer (Life Technologies). We used ABI Sequencing Analysis Software 5.2 program (Life Technologies) to standardize data and BioEdit (http://www.mbio.ncsu.edu/BioEdit/bioedit.html) to analyze sequences, which were subsequently BLAST-searched to identify the pathogen to the genus and species levels.

### Statistical Analyses

We calculated descriptive statistics (mean, median, range) for all continuous variables and compared differences between groups by using Student *t*-test or analysis of variance (ANOVA), or, in the case of nonnormal distribution, the Mann-Whitney rank-sum test or 1-way ANOVA on ranks. We quantified categorical variables by proportions and used Yates-corrected χ^2^ analysis to compare differences. We used GraphPad Prism version 6.0 (GraphPad Software, Inc., La Jolla, CA, USA) to perform all statistical computations. Significance was set at p<0.05.

## Results

We routinely screened 1,592 specimens for malaria during the enrollment periods of May 2006–April 2007 and February 2013–March 2014. Of those specimens, 165 (10.4%) were positive for *P. falciparum*, 93 (5.8%) for *P. vivax*, 20 (1.3%) for *P. ovale*, and 5 (0.3%) for *P. malariae*; 5 (0.3%) specimens had mixed *Plasmodium* infections. We subjected the remaining 1,308 malaria-negative specimens to the real-time PCR algorithm as described in the Methods to look for RNA- and DNA-based travel-acquired viruses. We did not assess the 165 malaria-positive specimens for co-infection with viral pathogens.

All assays detected control samples that had been confirmed positive. The dengue serotype–specific assay confirmed all types of DENV (DENV-1–4) without cross-reactivity among serotypes. Of the 1,308 malaria-negative specimens that were examined for multiple viral targets, only 1,304 could be tested as 4 were invalid (i.e., the internal reaction control β_2_ microglobulin could not be detected). A total of 262 (20.1%) of 1,304 specimens were positive (C_t_ <40) for at least 1 viral pathogen, 293 (22.5%) were positive for any detectable C_t_ and logarithmic amplification curve, and 31 (2.4%) had a C_t_ >40 with logarithmic amplification. DNA-based real-time PCR assays detected specimens positive for HSV-1 (n = 7, 0.54%), HSV-2 (n = 14, 1.1%), CMV (n = 4, 0.3%), and EBV (n = 194, 14.9%). RNA-based real-time RT-PCR assays detected CHIKV in 5 specimens (0.4%) and HAV in 12 (0.9%). real-time RT-PCR and subsequent Sanger sequencing identified DENV-positive specimens (n = 33, 2.5%) and untypeable flavivirus (n = 2, 0.15%). DENV subtyping by serotype-specific real-time RT-PCR and Sanger sequencing detected specimens positive for DENV-1 (n = 12, 0.92%), DENV-2 (n = 3, 0.23%), DENV-3 (n = 9, 0.69%), DENV-4 (n = 3, 0.23%), and untypeable DENV (n = 6, 0.46%). Nine (0.7%) specimens had mixed viral infections: EBV and DENV-1 (3 specimens), EBV and HAV (2 specimens), EBV and HSV-1 (2 specimens), DENV-1 and CHIKV (1 specimen), and CMV and HAV (1 specimen).

### Demographic Correlates of Detected Viral Pathogens

Of 1,304 malaria-negative EDTA-containing specimens tested for viral pathogens, 656 (50.3%) were from men and 621 (47.6%) from women; patient sex was unknown for 23 (2.1%) specimens. The distribution of cases between male and female patients was roughly the same: of the 262 specimens that were positive for at least 1 of the tested viruses, 132 (50.4%) were from male and 124 (47.3%) from female patients (p = 0.10). The lack of sex bias was observed across each assay. The average age of febrile returned travelers was 39.7 ± 23.1 years (median 39.1, range 10 months–90.6 years). Mean ages of patients infected with different viral pathogens were 23.0 ± 24.5 years for HSV-1, 36.5 ± 24.4 years for HSV-2, 41.6 ± 24.2 years for EBV, 32.3 ± 17.5 years for CMV, 37.6 ± 14.9 years for DENV, 36.7 ± 17.1 years for any flavivirus, 41.8 ± 10.2 years for CHIKV; and 27.5 ± 20.5 years for HAV (p = 0.17). Travel history was noted for 129 (49.2%) of the patients from whom the 262 specimens with at least 1 viral pathogen were derived (Table 3).

**Table 3:** Region of travel for patients from Canada who had viral pathogen–positive specimens after returning ill from travel, May 2006–April 2007 and February 2013–March 2014*

Viral pathogen	Total no. pathogens detected	No. (%) specimens with patient travel history noted	Region of travel, no. patients
HSV			
HSV-1	7	4 (57.1)	Afghanistan, 1; Caribbean, 1; Haiti, 1; Hawaii, 1
HSV-2	14	6 (42.9)	Dominican Republic, 3; India, 1; Sri Lanka, 1; Venezuela, 1
EBV	194	96 (47.7)	Indian subcontinent, 34; Caribbean, 21; Africa, 17; South America, 7; Central America, 5; Southeast Asia, 5; North America, 4; Middle East, 3
CMV	4	2 (50.0)	Pakistan, 1; South America, 1
Chikungunya virus	5	3 (60.0)	Barbados, 1; Philippines, 1; South Africa, 1
Dengue virus, serotype	33	20 (60.6)	
DENV-1	12	10 (83.3)	Barbados, 4; Cambodia, 1; Caribbean, 1; Dominican Republic, 1; India, 1; Sri Lanka, 1; Trinidad, 1
DENV-2	3	3 (100)	Bangladesh, 1; Cuba, 1; Tanzania, 1
DENV-3	9	2 (22.2)	Indonesia, 1; Philippines/Vietnam, 1
DENV-4	3	2 (66.7)	Vietnam, 2
Untypeable dengue	6	3 (50.0)	Guyana, 1; Malaysia, 1; Yemen, 1
Untypeable flavivirus	2	1 (50.0)	Barbados, 1
HAV	12	4 (33.3)	Pakistan, 3; Bangladesh, 1

*Data are for 129 (49.2%) of 262 EDTA-containing specimens. CMV, cytomegalovirus; DENV-1–4, dengue virus type 1–4; EBV, Epstein-Barr virus; HAV, hepatitis A virus; HSV-1 and 2, herpes simplex 1 and 2 virus.

## Discussion

We document common viral etiologies of fever in returned travelers by highly sensitive real-time PCR assays in >20% of malaria-negative specimens collected prospectively at a reference laboratory. At present, real-time PCR remains the best method for detecting viral pathogens causing acute illness, especially when low virus load is present in peripheral blood (*29*). In addition to its high sensitivity (and low limit of detection), real-time PCR offers the advantage of a fast turnaround time, operator independence, and the potential for high-throughput screening, testing, and detection; thus, it is a favored diagnostic tool, especially for detecting viruses that lead to transient and early viremia.

The lack of an etiologic diagnosis for up to 25% of febrile returning travelers is probably a result of insensitive diagnostic techniques (e.g., blood culture for detection of *Salmonella* Typhi), serologic testing that is performed outside the optimal acute and convalescent-phase window, and etiologies for which no readily available diagnostic test exists (e.g., novel and emerging flaviviruses, such as Zika virus). In addition, most testing done for the investigation of posttravel fever relies on single-pathogen detection assays, meaning that if a clinician fails to consider a particular virus in the differential diagnosis, testing for that virus will not automatically occur.

We noted high positivity rates for human herpesviruses in this study. Ninety-five percent of the human population is exposed to EBV and CMV by the age of 40, and the infection is often asymptomatic (*30*). Infection with HSV-1 and HSV-2 is not as common, however; the prevalence of infection within a population is 70%–100% (HSV-1) and 6%–50% (HSV-2) by 40 years of age (*30*). These herpesviruses replicate both lytically and latently; most infections are latent in the nervous system and lymphoid tissue. The herpesvirus positivity rates in our study ranged from 0.3% (HSV-1 and CMV) to 14.8% (EBV), numbers that are too low to reflect baseline prevalence rates in the general population and isolated latent infection. Rather, their detection probably reflects acquisition during travel or reactivation, either at home or abroad, in the setting of another antecedent illness. Another possibility would be primary infection before travel, with manifestation during or after travel. Indeed, all but 1 of 9 co-infections detected in this study involved at least 1 human herpesvirus, suggesting that primary acquisition of 1 virus precipitated secondary reactivation of another. Human herpesviruses can all cause a mononucleosis-like syndrome characterized by fever, malaise, lymphadenopathy, and mild biochemical hepatitis, and this nonspecific syndrome has been found to occur in up to a quarter of febrile returned travelers (*2*). Further delineation of the role of human herpesviruses in imported fever warrants prospective investigation with clinical linkage.

Flaviviruses include >70 different arthropodborne species, such as DENV, yellow fever virus, West Nile virus, Japanese encephalitis virus, and tickborne encephalitis virus, which are differentiated into 3 virus groups: mosquitoborne, tickborne, and unknown vector (*26*). Next to EBV, DENV was the most frequently detected viral pathogen in this study. Although DENV is endemic to many countries of Africa, Southeast Asia, and the Western Pacific region, where 50–100 million cases are reported each year (*26*), it causes disease that is increasingly imported from popular tourist destinations in the Americas, including the Caribbean. Thirty-percent of patients in this study with DENV-positive specimens had noted travel history to the Caribbean, and DENV-1 was particularly represented among these cases (7/12 cases). Our finding of only 2 undetermined flaviviruses is curious, given the broad *Flaviviridae* coverage of the selected primer and probe set (*26*). We cannot exclude the possibility of assay failure, but the possibility also exists of an importation of rare or novel flaviviruses acquired during travel.

CHIKV (family *Togaviridae*, genus *Alphavirus*) is also a mosquitoborne virus; the vectors, *Aedes albopictus* and *A. aegypti*, are known to carry DENV (*31*). Although dengue fever and chikungunya fever may have an identical early clinical manifestation, it is critical that the viral pathogen causing illness be identified in order to stratify patients by risk for complications arising from infection, including chronic arthropathy in the case of CHIKV infection and severe disease in the case of DENV infection. Serologically differentiating DENV from CHIKV may be challenging, given assay performance characteristics, timing of serologic testing, and known cross-reactivity of DENV IgG with that of other flaviviruses, including yellow fever vaccine virus (*31*). In addition, in the case of CHIKV, reliance on assays based on IgM only may lead to underdiagnosis during the convalescent phase of infection (*11*). We assume that enrollment of specimens further into 2014 and 2015 would have translated into much higher numbers of cases, given the maturation of the CHIKV outbreak in the Caribbean and Latin America.

Hepatitis A is one of the most common vaccine-preventable diseases, but the level of pretravel vaccination continues to be suboptimal among persons visiting HAV-endemic countries (*32*). In a study conducted with travelers from Sweden, the risk for travel-associated HAV infection was 0.67 cases per 100,000 travelers (*32*). The risk for infection is high for travelers to South America, Africa, and the Indian subcontinent; in our study, all HAV-positive patients with travel history noted had traveled to Bangladesh or Pakistan. That 0.9% of specimens were positive for HAV in this study underscores the remaining gap in pretravel vaccination.

Our analysis had several limitations. First, we lacked clinical linkage of specimens; thus, in each case, the exact clinical syndrome that prompted malaria screening is unknown. Given that malaria is not endemic to Canada and that persons who warrant malaria screening are likely unwell, we were reasonably confident that all included specimens would be from ill persons with a travel history. Second, we limited our viral detection algorithm to those viruses or virus families that are known to commonly cause fever in returned travelers. Therefore, our results probably represent an underestimate of virus burden in this cohort of specimens. For instance, we did not look for HIV, a virus that is an infrequent cause of fever in returned travelers, nor did we search for the agents of viral hemorrhagic fever, which, before the 2013–2015 Ebola virus crisis in western Africa, were exported less than a handful of times by travelers. We also did not search for influenza or other predominantly respiratory viruses due to the absence of a true viremic stage in the infection process, although these agents are well recognized to cause fever in returned travelers. Third, we relied exclusively on the specimen requisition forms for acquiring travel histories for patients, and forms for less than half of the patients included this information. The lack of travel histories may have biased our interpretation of the geographic representation of detected pathogens. Fourth, we only performed our diagnostic assays on EDTA-containing blood, the sensitivity of which would be affected by the level of viremia and the timing of initial malaria diagnostics. For instance, viremia in CHIKV and DENV infection is short-lived, so the prevalence of DENV and CHIKV might have been higher had serologic evaluations been used. Fifth, our reported rates of viral illnesses could represent an underestimate if the initial malaria diagnostic specimen came from a patient who was afebrile and without risk factors for travel-acquired illness. We believe that this possibility is low because malaria testing is not routine and, if anything, is underordered in areas where malaria is not endemic. Last, as mentioned, we were unable to differentiate primary infection from reactivation disease in the specimens positive for human herpesviruses.

We documented infections with common and emerging viral pathogens, including 12 cases of acute hepatitis A, which is vaccine preventable, in >20% of malaria-negative specimens from travelers who were ill when they returned to Canada. A substantial diagnostic gap remained in this group of specimens from febrile returned travelers, despite application of highly sensitive and family-level real-time PCR assays. Even in the setting of positive flavivirus and DENV real-time PCR, some viruses remained untypeable. Further elucidation of the specific etiology of fever in returned travelers should be undertaken with increasingly sophisticated diagnostics, such as next-generation sequencing, to better understand the full spectrum of pathogens causing this potentially life-threatening syndrome.
